# Factors associated with subchorionic hematoma formation in pregnancies achieved via assisted reproductive technologies

**DOI:** 10.1007/s10815-019-01684-7

**Published:** 2020-01-06

**Authors:** Brady T. West, Parviz K. Kavoussi, Kate C. Odenwald, Krista London, Caitlin L. Hunn, Shu-Hung Chen, John David Wininger, Melissa S. Gilkey, Keikhosrow M. Kavoussi, Shahryar K. Kavoussi

**Affiliations:** 1 Survey Research Center, Institute for Social Research, University of Michigan, Ann Arbor, MI 48109 USA; 2 Austin Fertility and Reproductive Medicine/Westlake IVF, 300 Beardsley Lane, Bldg B, Suite 200, Austin, TX 78746 USA

**Keywords:** Subchorionic hematoma, Subchorionic clot, IVF, In vitro fertilization, Assisted reproductive technologies

## Abstract

**Purpose:**

To determine if certain clinical and/or embryologic factors are independently associated with the increased prevalence of subchorionic hematoma (SCH) among pregnancies achieved via in vitro fertilization (IVF) with fresh embryo transfer (ET).

**Design:**

Retrospective chart review.

**Methods:**

In this retrospective study, data were abstracted from 210 autologous oocyte IVF clinical pregnancies that resulted from fresh ET at a single fertility center from January 2012 through December 2016. Clinical and embryology laboratory variables were analyzed as possible factors associated with the presence or absence of SCH in IVF pregnancies via bivariate associations and multivariable logistic regression analyses. Independent variables included prior uterine surgery versus no uterine surgery, peak estradiol, and progesterone levels, day 3 (*n* = 92) versus day 5 (*n* = 118) ET, and assisted hatching versus no assisted hatching. Among the day 5 ET subgroup of 118 patients, 117 had data for the variables inner cell mass (ICM) grading and trophectoderm (TE) because one day 5 ET was at the morula stage.

**Results:**

We found a significant bivariate association between TE grading and SCH, where cases with TE grade “A” were significantly less likely to have SCH compared with cases with grades “B” or “C.” This significant difference remained when adjusting for the other factors considered in a multivariable logistic regression model for the probability of SCH.

**Conclusions:**

The data analyzed here suggest that a less-advanced trophectoderm grade may be a potential factor that is associated with the presence of SCH in pregnancies achieved via IVF.

## Introduction

For patients who achieve clinical pregnancy via in vitro fertilization (IVF), several findings may adversely affect resulting pregnancies or be a source of distress for patients. One such finding, subchorionic hematoma (SCH), is found in 4 to 48% of pregnancies in general [1] and has been reported to be present more frequently in singleton IVF pregnancies than in singleton non-IVF pregnancies [2, 3]. It has been postulated that the elevated estradiol levels due to controlled ovarian hyperstimulation (COH) in IVF may predispose to a local endometrial environment more susceptible to SCH formation. SCH is diagnosed via transvaginal sonogram (TVS) either incidentally or during evaluation of uterine bleeding during early pregnancy. The presence and symptoms of SCH may cause varying degrees of distress as well as concern among clinicians and patients regarding the potential for adverse pregnancy outcomes. Reported adverse outcomes associated with SCH in non-IVF pregnancies include increased risk of pregnancy loss, abruption, preterm premature rupture of membranes (PPROM), and fetal growth restriction [4–8]. Recent publications have suggested that SCH before 14 weeks of gestation is neither associated with pregnancy loss before 20 weeks of gestation nor adverse pregnancy outcomes after 20 weeks of gestation [9, 10]. Moreover, a recent retrospective cohort study of 1097 IVF cases suggested that, although SCH was associated with lower birth weight in singleton gestations, the pregnancy loss rate was not increased among IVF/ICSI patients. Furthermore, the authors concluded that SCH may be associated with fresh embryo transfer (ET) [11].

Although the exact mechanism by which SCH form is uncertain, SCH may result from the partial detachment of the chorionic membrane from the decidual membrane [7]. Furthermore, it is thought that SCH formation is associated with events such as the rupture of blood vessels during the process of villous invasion into the endometrium, which may lead to abnormal placentation [4, 5, 7]. The aim of this study was to determine whether certain clinical and/or embryology factors are independently associated with the increased prevalence of SCH among pregnancies achieved via IVF and, if so, potentially suggest a different mechanism of action by which SCH form in IVF pregnancies.

## Materials and methods

In this retrospective study, data were reviewed from 210 IVF clinical pregnancies, defined as sonographic evidence of fetal pole with cardiac motion at 6–7 weeks of gestation that resulted from fresh ET at a single fertility center from January 2012 through December 2016. Some analyses were restricted to only cases with blastocyst stage embryo transfer, which were 117 of the aforementioned 210 clinical pregnancies. Clinical records and IVF laboratory records were reviewed. Exclusion criteria included autologous oocyte frozen embryo transfer (FET) cycles, donor oocyte cycles, donor embryo cycles, and cases that involved a gestational carrier. Due to the de-identified nature of the data collected, Institutional Review Board exemption was obtained from St. David’s Healthcare Institutional Review Board.

For the controlled ovarian hyperstimulation (COH) portion of the IVF process, individualized protocols based on patient age and AMH level were used, including the short gonadotropin releasing hormone (GnRH) agonist, GnRH antagonist, and GnRH agonist microdose flare protocol. All patients had daily baby aspirin as an adjunct to COH protocol. After ovarian stimulation and subsequent oocyte retrieval 35–36 h after human chorionic gonadotropin (hCG) administration, either IVF or intracytoplasmic sperm injection (ICSI) was performed and embryo transfer was performed on day 3 or day 5, based on embryo quality, and in accordance with American Society for Reproductive Medicine/Society for Assisted Reproductive Technology (ASRM/SART) guidelines for number of embryos to be transferred [12]. Supernumerary good quality blastocysts were cryopreserved on day 5 or day 6.

If a gestational sac with fetal pole and sonographic evidence of fetal heart activity was established by TVS for a given patient at 6–7 weeks of gestation, then follow-up TVS was routinely performed at 2-week intervals until 10–11 weeks of gestation if the pregnancy continued to progress. Some patients had additional ultrasound assessments between 6 and 11 weeks of gestation due to vaginal bleeding.

Selected clinical measures as well as embryology laboratory techniques and variables were analyzed as possible factors associated with the presence or absence of SCH in IVF pregnancies. These measures are described in more detail in Table 1.

**Table 1:** Descriptions of clinical and embryology measures analyzed in this study as potential factors associated with the presence of SCH in IVF pregnancies

Variable	Value labels	Percentages/means
SCH	1 = yes 0 = no	30.95% (65/210) 69.05% (145/210)
Day of embryo transfer	3 = day 3 5 = day 5	43.81% (92/210) 56.19% (118/210)
SACS	1 = one sac 2 = two sacs 3 = 3 sacs	68.57% (144/210) 30.95% (65/210) 0.48% (1/210)
TE*	1 = grade A 0 = grade B or C	52.99% (62/117) 47.01% (55/117)
Prior uterine surgery	1 = yes 0 = no	20.00% (42/210) 80.00% (168/210)
LAH	1 = yes 0 = no	57.62% (121/210) 42.38% (89/210)
Peak E2	Continuous	Mean = 2089.51, SD = 965.25, *n* = 208
Final P4	Continuous	Mean = 0.82, SD = 0.35, *n* = 200
ICSI	1 = yes 0 = no	91.90% (193/210) 8.10% (17/210)
Mature oocytes retrieved	Continuous	Mean = 10.56, SD = 5.36, *n* = 210
1-cell embryos	Continuous	Mean = 8.27, SD = 4.39, n = 210
Number of embryos transferred	Continuous	Mean = 2.11, SD = 0.70, n = 210

*TE frequencies are restricted to the 117 cases where this grading was performed

*SCH*, subchorionic hematoma; *TE*, trophectoderm; *LAH*, laser-assisted hatching; *E2*, estradiol; *P4*, progesterone; *ICSI*, intracytoplasmic sperm injection

**Fig. 1:**
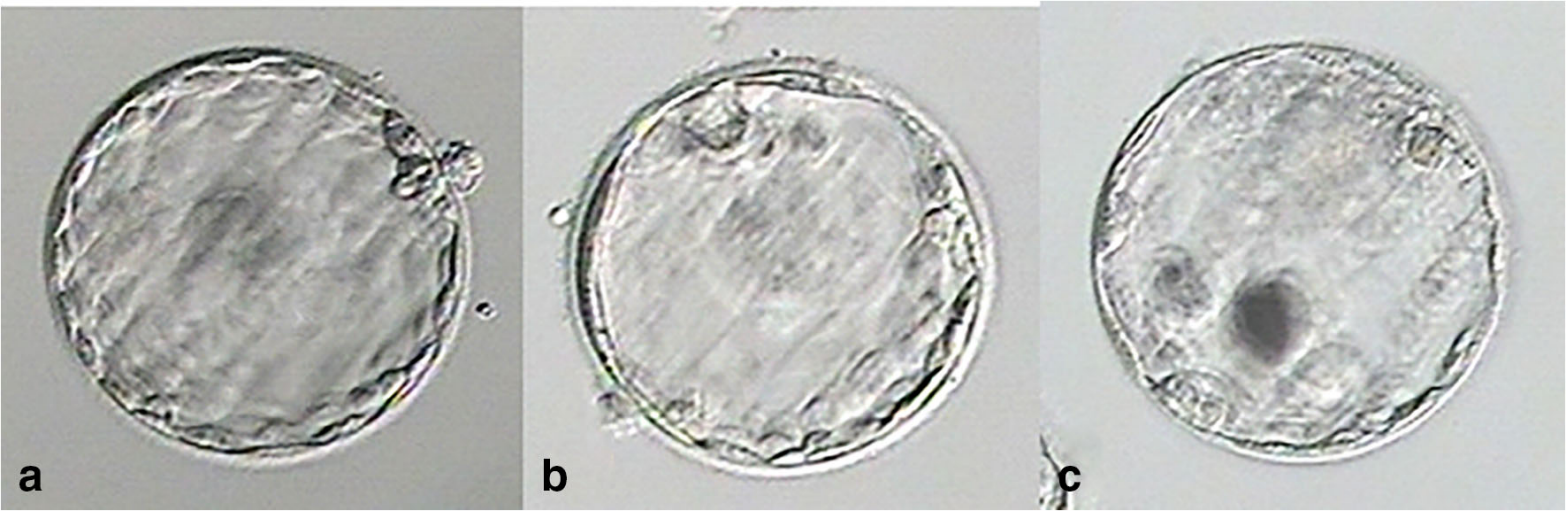
Examples of blastocyst trophectoderm (TE) grading A, B, and C

The bivariate associations of the factors described in Table 1 with SCH were tested using two-variable chi-square tests and independent samples *t* tests, and multivariable associations were examined via logistic regression analysis. Cases with missing data on any of the variables were dropped from the analyses. Independent variables considered in the logistic regression analysis included prior uterine surgery versus no uterine surgery, peak estradiol, progesterone levels (Peak E2 and Final P4), use of intracytoplasmic sperm injection (ICSI) or no ICSI, number of metaphase II oocytes at retrieval (M2), number of one-cell two pronuclear embryos (2PN), number of embryos transferred, day 3 versus day 5 embryo transfer, assisted hatching versus no laser assisted hatching (LAH), inner cell mass (ICM) grading, and trophectoderm (TE) grading. When more than one blastocyst was transferred, the less-advanced stage was used for analysis. The embryology team used the Gardner blastocyst grading system [13] uniformly during the study period, and examples of TE grades A, B, and C are shown in Fig. 1. The number of patients analyzed in the multivariable analyses depended on the number of cases with complete data on all of the variables considered in the analysis (i.e., listwise deletion was employed).

## Results

A significant bivariate association was found between TE and SCH, with 41.82% of pregnancies having TE equal to B or C having SCH, compared with only 20.97% of pregnancies having TE equal to A [chi-square(1) = 5.95, *p* = 0.015, *n* = 117]. There was also weak evidence of a significant bivariate association between SCH and final P4 (peak progesterone level on day of trigger), with the SCH group having a lower mean final P4 value (*p* = 0.043).

In a multivariable logistic regression model without TE as a predictor (*n* = 199, after dropping the one case with 3 sacs), none of the variables in Table 1 were found to be independent predictors when adjusting for the relationships of other variables with SCH. In a multivariable logistic regression model fitted to only those cases where TE grading was performed (*n* = 113 after dropping cases with missing data), the TE grade was found to be the only significant predictor of SCH, where pregnancies receiving grade A had 75% reduced odds of having SCH present when adjusting for the other factors (adjusted odds ratio = 0.25, 95% CI = 0.09–0.68, *p* < 0.01). No other factors were found to be significant predictors of SCH in this model, and the model was found to have a good fit based on the Hosmer-Lemeshow goodness-of-fit test (*p* = 0.11). See Table 2 for the estimated odds ratios in this model.

**Table 2:** Estimates of adjusted odds ratios in a multivariable logistic regression model fitted to the SCH data for cases with no missing data where TE grading was performed (*n* = 113)

Predictor	Adjusted odds ratio	95% confidence interval
SACS		
1	Reference	
2	0.93	0.35–2.48
TE		
A	0.25	0.09–0.68**
B or C	Reference	
Prior uterine surgery		
Yes	0.62	0.20–1.87
No	Reference	
LAH		
Yes	1.59	0.64–3.95
No	Reference	
Peak E2 (in thousands)	0.58	0.31–1.08
Final P4	0.35	0.08–1.42
ICSI		
Yes	2.28	0.39–13.33
No	Reference	
Mature oocytes retrieved	1.00	0.84–1.20
1-cell embryos	0.95	0.78–1.17
Number of embryos transferred	0.78	0.29–2.05

*p < 0.05; ** *p* < 0.01; Hosmer-Lemeshow goodness-of-fit test *p* value = 0.11

In terms of pregnancy outcomes, there was no difference in spontaneous miscarriage rate between IVF pregnancies with SCH (18.5%; 12/65) and those without SCH (15.2%; 22/145; *p* value = 0.69).

## Discussion

The aim of this study was to determine whether certain clinical and/or embryology laboratory factors were independently associated with the increased prevalence of SCH in pregnancies that result from fresh IVF-ET cases, thereby suggesting a possible mechanism of action of SCH formation in IVF pregnancies. The data analyzed suggest that a less-advanced trophectoderm grade (B or C) increases the odds of SCH presence in IVF pregnancies, and this association remained robust in a multivariable logistic regression model adjusting for age, uterine surgery, peak estradiol level on day of trigger, peak progesterone level on day of trigger, ICSI or no ICSI, number of M2 oocytes, number of 2PN embryos, number of embryos transferred, laser-assisted hatching, and inner cell mass. These findings are notable, given that only 113 IVF pregnancies resulting from day 5 ET across these 4 years had available data present on all variables of interest, limiting our statistical power. The findings suggest that this is a notable relationship with a relatively large effect size.

Limitations of our study include the potential biases that are inherent with a retrospective analysis as well as the sample sizes of groups with day 5 ET. SCH morphology was not specifically described in all ultrasound reports; therefore, this information was not available to be included in this study. In addition, baby aspirin was used as part of the protocol, which may be associated with the presence of SCH [14]. Although it is possible that the use of baby aspirin may increase the incidence of SCH in cases of lesser TE cellularity as opposed to aspirin-free protocols, the percentage of IVF pregnancies which had SCH was lower in our study (31%, 65/210) than IVF pregnancies in the study by Truong et al. (49%, 65/132) [14]. Although the use of baby aspirin is a potential confounding factor, since all IVF patients in our study had baby aspirin as part of their protocol, this factor was uniform among the study and control group subjects. Another study limitation was the inclusion of double embryo transfer cases where one embryo had a less-advanced TE than the other. In such cases, we chose the morphologic assessment of the less-advanced TE for analysis. In addition, since pregnant patients transitioned care to their general obstetrician-gynecologist after 10–11 weeks of gestation, duration of SCH for all cases as well as complete obstetric and delivery complication data were not available in our study database.

Although SCH has not been found to be associated with increased spontaneous miscarriage rates specifically among pregnancies achieved via ART, the common symptom of first trimester bleeding associated with SCH is a source of distress and concern for patients and clinicians. In the present study, there was no difference in spontaneous miscarriage rate between IVF pregnancies with SCH and those without SCH (18.5% vs 15.2%; *p* value = 0.69), consistent with findings in the prior publication by Zhou et al. [11]. There are data indicating a relatively high incidence of SCH among pregnancies conceived via IVF, and the findings in our study suggest that, among blastocyst transfers, SCH is more likely to be seen in pregnancies for which a blastocyst with a less-advanced trophectoderm is transferred to the uterus. TE grading has been shown to be associated with other IVF outcomes in previous studies [15–17]; however, this is the first study to suggest an association between TE grading and the presence of SCH in pregnancies resulting from IVF. Larger future studies with only single embryo transfer would be helpful in further validating the findings of our study, with the potential clinical application being the selection of blastocysts with trophectoderm A grading, if available, in order to try to minimize the incidence of SCH and its associated symptoms and stress for patients and clinicians.
